# The role of proopiomelanocortin (POMC) neurones in feeding behaviour

**DOI:** 10.1186/1743-7075-4-18

**Published:** 2007-09-01

**Authors:** George WM Millington

**Affiliations:** 1 Division of Medicine, Norfolk and Norwich University Hospital, Colney Lane, Norwich, NR4 7UZ, UK

## Abstract

The precursor protein, proopiomelanocortin (POMC), produces many biologically active peptides via a series of enzymatic steps in a tissue-specific manner, yielding the melanocyte-stimulating hormones (MSHs), corticotrophin (ACTH) and β-endorphin. The MSHs and ACTH bind to the extracellular G-protein coupled melanocortin receptors (MCRs) of which there are five subtypes. The MC3R and MC4R show widespread expression in the central nervous system (CNS), whilst there is low level expression of MC1R and MC5R. In the CNS, cell bodies for POMC are mainly located in the arcuate nucleus of the hypothalamus and the nucleus tractus solitarius of the brainstem. Both of these areas have well defined functions relating to appetite and food intake. Mouse knockouts (ko) for *pomc*, *mc4r *and *mc3r *all show an obese phenotype, as do humans expressing mutations of *POMC *and *MC4R*. Recently, human subjects with specific mutations in *β-MSH *have been found to be obese too, as have mice with engineered β-endorphin deficiency. The CNS POMC system has other functions, including regulation of sexual behaviour, lactation, the reproductive cycle and possibly central cardiovascular control. However, this review will focus on feeding behaviour and link it in with the neuroanatomy of the POMC neurones in the hypothalamus and brainstem.

## Background

Over the last decade, there has been much research on the role of hypothalamic POMC neurones, with regard to appetite. However, POMC products (the MSHs, ACTH and β-endorphin) also have important roles in the skin, stress response, immune system and sexual function [1-6]. Experimental evidence suggests that POMC neurones form an integral part of the central melanocortin system regulating feeding behaviour. One or more of the melanocortins and β-endorphin, released from these POMC neurones, may be involved in this regulation. When specific genes in the CNS melanocortin system (*POMC*, *PC1*, *MC4R *and *MC3R*) are found mutated in either humans or rodents, this results in an obese phenotype [7-9].

There have been two recent excellent reviews regarding the electrophysiology of feeding circuits and the neuroanatomy of POMC [10,11]. However, the focus of this review is the function of POMC projections in the CNS, which originate from the arcuate nucleus of the hypothalamus and the brainstem and their role in appetite and feeding control. The review begins with a brief overview of feeding systems and specifically the role of leptin as a major peripheral signal to the POMC arcuate neurones. Then, the basic biology of POMC and melanocortin receptors is discussed, together with some observations from human genetic studies. The next section deals with the two groups of POMC neurones in the arcuate nucleus and brainstem, as well as their melanocortin and β-endorphin products, MC4R, MC3R and feeding. This is set in the context of other neurotransmitter systems that have been shown to interact with the melanocortin system in these two brain regions. Then other brain regions that might be targets for POMC mediated effects are discussed, namely the paraventricular nucleus (PVN), lateral hypothalamus, dorsomedial nucleus (DMH), supraoptic nucleus (SON), ventromedial nucleus (VMH), periventricular nucleus, nucleus accumbens and amygdala. As far as possible, other interacting neurotransmitters are discussed in the sections relating to where their cell bodies or projections are located.

### Feeding and energy homeostasis

Feeding is primarily a response to habitually entrained rhythms, including circadian rhythms. Feeding behaviour is regulated by a system with the hypothalamus at the centre, where how much we eat is a response to an internal energy status [12]. There are complex but integrated interconnections between the hypothalamic nuclei that maintain energy homeostasis through regulation of food intake and energy expenditure [13-16]. One of the key components of the hypothalamic system is the POMC neurones of the arcuate nucleus [10,11]. One of the main longer term peripheral signals is the hormone leptin [10,11].

### Leptin and hypothalamic regulation

The site of leptin's action is the mediobasal hypothalamus, principally the hypothalamic arcuate nucleus, via the long form of the leptin receptor (LEPRB) that influences the activities of two separate groups of neurones with opposing feeding functions. These include POMC neurones that co-express CART and AgRP neurones that co-express NPY. These arcuate POMC neurones have processes close to the fenestrated capillaries of the median eminence and can thus be targeted by hormones such as leptin in the circulation [17]. POMC neurones in the arcuate nucleus, which express LEPRB, are activated by leptin. They project to the DMH, PVN and lateral hypothalamus [10,11]. Leptin may thus be the signal linking peripheral energy stores with POMC signalling activity in the hypothalamus. LEPRB is also expressed in many other hypothalamic nuclei that may have a role in appetite. These include the DMH, VMH, PVN, lateral hypothalamic area, periventricular nucleus and SON [18,19]. It must be remembered that not all mammalian hypothalamic POMC neurones express leptin receptors [20,21], suggesting the existence of a leptin-unrelated melanocortin signalling system too.

## Proopiomelanocortin (POMC) genetics and its post-translational modification

**Figure 1:**
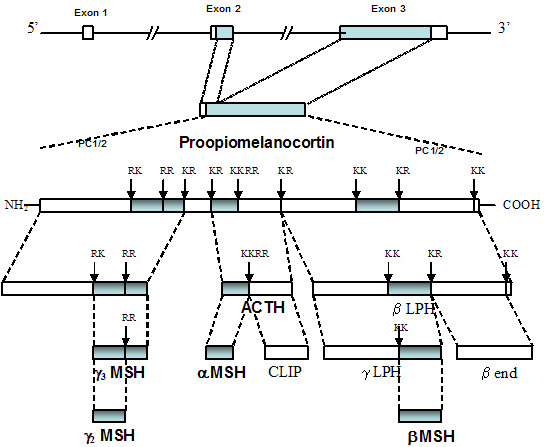
**Gene structure and post-translational processing of proopiomelanocortin (POMC)**. POMC in mammals consists of 3 exons, of which exons 2 and 3 are translated. Prohormone convertases 1 and 2 (PC1/2) break the parent POMC peptide into successively smaller peptides by cleavage at paired dibasic amino acid residues consisting of lysine (K) and/or arginine (R). The final products are generated in a tissue specific manner, for example α-MSH and ACTH are not produced by the same cells in the pituitary. They also involve additional enzymatic post translational modifications, such as the acetylation of α-MSH. The final products include the melanocortins (MSHs and ACTH), β-endorphin (β-end) and corticotrophin-like intermediate peptide (CLIP). There are intermediate peptides whose biological function remains unclear, such as β and γ lipotrophins (β-LPH, γ-LPH).

POMC is the precursor of the MSHs and adrenocorticotrophin (ACTH), as well as β-endorphin (Figure 1). The MSHs and ACTH are collectively known as melanocortins. All sequenced mammalian *POMC *genes consist of three exons, interspersed by large introns (Figure 1; [22,23]). *pomc *mRNA is synthesised in the pituitary, the arcuate nucleus, the nucleus tractus solitarius of the brainstem (NTS) and several peripheral tissues. This mRNA forms a single protein, which passes to the Golgi bodies. The signal peptide sequence directs it into secretory granules where post-translational processing yields many peptides by successive, cell-specific, enzymatic modifications (Figure 1; [24-27]). Cleavage by the propeptide convertases PC1 and PC2 occurs at specific pairs of basic residues, Lys and Arg [28-30]. PC1 alone is expressed in the pituitary corticotrophs and cleaves POMC, producing the NH_2_-terminal peptide (NT), joining peptide (JP), ACTH, β-lipotrophin (β-LPH) and β-endorphin. Melanotrophs generate PC1, PC2, carboxypeptidase E (CPE)), amidating and *N*-acetylating enzymes to produce α-MSH and β-endorphin [28,29,31,32]. PC2 cleaves the first 14 amino acidsof ACTH to generate ACTH (1–14) OH. PC2 cleaves β-LPH to produce β-MSH and β-endorphin. The ACTH (1–14) OH, after COOH-terminal amidation, produces desacetyl-α-MSH, a step required for biologic activity. α-*N*-acetylation of the NT enhances the action of α-MSH and inhibits the action of β-endorphin [33]. *O*-acetylation of α-MSH forms *N*,*O*-diacetyl-α-MSH. PC2 ko mice produce no α-MSH at all [34]. PC1 or PC2 may produce the γ-MSHs but the mechanism is not clear [28,29]. Paired amino-acid converting enzyme 4 (PACE4) colocalises with POMC in the hypothalamus [35]. Both PACE4 and POMC converting enzyme (PCE), a secretory vesicle aspartyl protease, can cleave POMC in vitro [35,36]. How these additional enzymes interact with PC1 and PC2 is not clear at present. The subject of POMC processing in the hypothalamus has been reviewed in more detail recently [37].

### The genetic pathology of the mammalian melanocortin system

*POMC *deficiency in mice and humans and *PC1 *homozygous mutations in humans are characterised by adrenal failure, early-onset obesity and, with *POMC *mutations, altered pigmentation and tall stature [7,9,38]. Similarly, *cpe *ko mice show obesity, as well as a number of other endocrine defects [27]. Subjects with the human genetic obesity disorder, Prader-Willi syndrome, have reduced levels of PC2 in their hypothalami on immunocytochemical assessment [39].

A point mutation in the cleavage site between β-MSH and β-endorphin (Figure 1) forms an aberrant fusion protein, which causes obesity in humans, perhaps by altering central melanocortin signalling in the CNS [40]. Heterozygous mutations in *MC4R *also cause obesity and tall stature in humans [7]. Recent data suggests that β-MSH may be the important POMC product in inhibiting feeding centrally in humans [41], but not rodents, as rodents do not produce β-MSH physiologically [7,42].

Mendelian human *POMC *disorders are very rare [43], but there is linkage between *POMC *and other obesity related traits, suggesting that it may form part of a common genetic obesity predisposition [44,45]. These findings suggest a clinically relevant role for POMC within the feeding circuits in the CNS.

## Melanocortin receptors (MCRs)

The melanocortins all contain the amino acid sequence His-Phe-Arg-Trp, required for receptor occupancy. The MCRs are part of the G-protein coupled receptor family. Five MCRs are cloned, the MC1R, MC2R, MC3R, MC4R and MC5R [46]. MC1R is predominantly the α-MSH receptor and MC2R the ACTH receptor. In radioligand binding studies, β-MSH shows more affinity to the MC4R than α-MSH, with γ-MSH having the least affinity. γ-MSH binds to the MC3R more than α- or β-MSH. MCR signalling activates adenyl cyclase, leading to accumulation of cAMP, as well as protein kinase C (PKC) and diacylglycerol (DAG) downstream [1,46].

## POMC neurones and the arcuate nucleus

The arcuate nucleus lies at the base of the hypothalamus. It is responsive to peripheral stimuli such as leptin. Leptin is essential in the neonatal phase for the development of neuronal projections from the arcuate nucleus to the DMH, PVN and lateral hypothalamus in succession, as shown by DiI (1,1'-dioctadecyl-3,3,3',3'-tetramethylindocarbocyanine perchlorate) axonal labelling [47].

### POMC/CART and NPY/AgRP neuronal pathways from the arcuate nucleus

There are two main neuronal populations located in the arcuate nucleus regulating appetite. There is evidence from both gene and protein expression studies that the satiety neurones produce both POMC and cocaine and amphetamine regulated transcript (CART) [48]. Food restriction reduces hypothalamic *pomc *mRNA expression [49], whereas hypothalamic *pomc *mRNA expression is increased in overfed rats [50]. The orexigenic neurones contain NPY and agouti-related peptide (AgRP). Fasting activates mRNA expression in hypothalamic NPY neurones [51]. NPY is thought to be one of the strongest stimuli to feeding [51] perhaps partly acting by inhibiting arcuate nucleus *pomc *mRNA expression via the Y_2 _receptor [52]. The major function of AgRP is to stimulate feeding by antagonising melanocortins at the MC3R and MC4R in the hypothalamus [53]. Selective ablation of NPY/AgRP-expressing neurones in adult mice results in acute reduction of feeding [54]. *AgRP *polymorphisms are associated with inherited leanness in humans [55]. In the parvocellular division of the paraventricular nucleus, close apposition of the perikarya of POMC/CART neurones and NPY/AgRP-containing terminals is seen. CART arcuate nucleus neurones markedly inhibit NPY-induced feeding in fasted and normal rats [56].

Both the POMC/CART and NPY/AgRP pathways express LEPRB and project to the leptin-dependent regions in the DMH, PVN and lateral hypothalamus (Table 1).  Leptin has opposite effects on each pathway with regard to feeding, facilitating transmission in the POMC/CART system and inhibiting the NPY/AgRP neurones, when measure by a system using fluorescent protein (GFP)-tagged POMC (red) and NPY (green) neurones  [57]. Surgical disruption of the arcuate nucleus to PVN connection in rats results in increased food intake and weight gain, including body fat, implying that the balance of the background anorexigenic effects of POMC/CART neurones is greater than the orexigenic effects of the NPY/AgRP neurones [58]. It must be born in mind that lesioning experiments are likely to disrupt many neuronal connections and their interpretation requires some caution.

**Table 1:** A summary of the experimental evidence for the presence of POMC axonal projections involved in feeding behaviour (axonal labelling vs immunocytochemistry – references indicated numerically)

**Arcuate POMC projections:**	**Axonal labelling**	**Immunocytochemistry**
**Arcuate nucleus**	57	48, 62, 93, 119
**Brainstem**	91	91
**PVN**	-	62, 93
**Lateral hypothalamus**	-	93
**DMH**	160	93
**SON**	-	93
**VMH**	-	93
**Periventricular nucleus**	-	93, 172
**Nucleus accumbens**	-	93
**Amygdala**	-	93

Using *ob/ob *mice expressing GFP on both POMC and NPY neurones in the arcuate nucleus, leptin-deficiency has been shown to increase both the number of excitatory synapses to NPY/AgRP neurones and the number of inhibitory synapses to POMC/CART neurones. When leptin is given to the *ob/ob *mice, synaptic numbers revert to the wild type pattern within 6 hours [57]. Fasting leads to an increase in the action potential of the arcuate nucleus NPY/AgRP neurones in normal mice, but this is not seen in *ob/ob *or *db/db *strains lacking central leptin signalling [59]. This leptin-induced neuronal plasticity may relate to its role as a signal of fat stores. Melanotan II (MTII), an MC3R/MC4R agonist reduces the orexigenic and adipogenic effects of NPY, but does not alter NPY-induced suppression of the reproductive and growth neuroendocrine axes [60]. A large proportion of NPY neurones in the rat hypothalamus express *mc3r *mRNA while a much lower number of NPY neurones express *mc4r *mRNA, suggesting that POMC neurones may directly modulate the activity of the hypothalamic NPY system too, mainly through activation of MC3R [61].

A subpopulation of arcuate nucleus NPY/AgRP/GABA-producing neurones, identified immunocytochemically, project to the PVN and send inhibitory GABA collaterals to arcuate nucleus POMC neurones that express GFP [62].  GABA blocks the anorexic effect of icv α-MSH, whereas a GABA antagonist increases the anorexia [63]. Thus, GABA facilitates the feeding effect of NPY at target sites in the PVN, by blocking opposing POMC transmission. However, in *ob/ob *mice (who congenitally lack leptin, leading to obesity), leptin deficiency exaggerates the direct inhibitory effect of NPY neurones on POMC neurones in the arcuate nucleus, an effect independent of the actions of GABA [64]. Also, approximately one-third of POMC/CART neurones express glutamic acid decarboxylase (GAD) mRNA [47]. GAD is the enzyme necessary to produce GABA, although GABA is not thought to inhibit the NPY/AgRP neurones and its exact function is unknown at present. It may be involved more in the extra-hypothalamic projections of POMC [65]. There is recent immunocytochemical evidence that hypothalamic POMC neurones express cholinergic fibres too [66]. Studies utilising immuno-electron microscopy, immunocytochemistry and in-situ hybridisation show that both the NPY/AgRP nerve fibres and the CART/POMC neurones are innervated by glutaminergic fibres too [67,68]. This links the control of feeding into wider feedback systems, for example glutamate neurones connect to the VMH, DMH and lateral hypothalamus, the other main feeding areas in the hypothalamus [67,68].

### AgRP – more than an antagonist of MC4R and MC3R?

The melanocortins have two endogenous antagonists, agouti and AgRP, that show some subtype selectivity as MCR antagonists. AgRP only acts on the CNS receptors, MC4R and MC3R (Table 2; [69]). Both AgRP and the MSHs can bind to MC3R and MC4R either presynaptically or postsynaptically [10,11]. Like POMC, AgRP may undergo post-translational processing [70]. Both agouti and AgRP function as inverse agonists in vitro, so they may regulate their respective MCRs in vivo, even in the absence of melanocortins [71].

**Table 2:** Melanocortin receptors and their endogenous antagonists

**Melanocortin receptor**	**Endogenous antagonist**
MC1R	Agouti
MC2R	Agouti
MC3R	Agouti, AgRP
MC4R	Agouti, AgRP
MC5R	?

In hamsters, central administration of AgRP increases food hoarding more than food intake [72]. Does this suggest species differences in the response to AgRP, or perhaps actions other than pure antagonism of the melanocortin anorexic signal? AgRP_83–132 _has been shown to have effects in the *mc4r *ko mouse, which could be attributable to MC3R antagonism, or perhaps as yet unknown effects on a different receptor system [73].

Perhaps AgRP neuronal activity is more directly linked to metabolic change than POMC neuronal activity? In the *ob/ob *mouse, *agrp *gene expression is increased 5–10 fold compared with wild type [74,75], whereas the absence of leptin only reduces *pomc *gene expression by approximately two-fold [76]. Others have found that there is virtually no change in hypothalamic *pomc *expression in either food restricted, fasted or leptin resistant (Zucker) rats versus controls [70]. Overall this gives a picture of a rather tonic anorexic signal from the POMC/CART neurones, perhaps with a more finely tuned and responsive orexigenic signal from the AgRP/NPY system? AgRP, its interactions with the melanocortin system and other roles has been well reviewed recently [70].

### Peptide YY and POMC

Peptide YY (PYY) and NPY are part of the same peptide family, acting via the Y-group of receptors. After a meal, the gut releases PYY_3–36 _into the circulation, which crosses the BBB and reduces food intake by suppressing NPY release and increasing POMC release in the arcuate nucleus [77-80]. PYY_3–36 _inhibits the action potential firing activity of arcuate POMC neurones, acting through postsynaptic Y2 receptors [81]. However, *mc4r *ko, *pomc *ko and normal mice have similar anorexia with PYY, suggesting it can act independently of melanocortins too [9,82]. This may seem paradoxical, but could relate to differing development of the hypothalamic NPY/PYY system in the absence of *pomc *or *mc4r *from birth.

### VGF, lethal yellow and POMC

VGF (non-acronymic) is a hypothalamic neuropeptide coexpressed in POMC and NPY neurones, identified by immunocytochemistry, in the arcuate nucleus [83]. Lethal yellow is an inbred obese mouse strain, resulting from overexpression of agouti, which then has excess AgRP-like actions in the CNS [69]. *vgf *ko mice are lean with normal food intake and increased oxygen consumption and locomotor activity at rest [83]. Crossing the *vgf *ko mouse with *ob/ob *and lethal yellow mutants, improves the obesity of the *ob/ob *mouse and prevents the lethal yellow obese phenotype. This suggests that the actions of VGF are downstream of AgRP and the melanocortins [83]. Arcuate nucleus *vgf *mRNA expression is increased in hamsters in experimental short photoperiod situations mimicking winter, suggesting a feeding-related hibernation function for VGF [84].

### Galanin and POMC

Cells in the arcuate nucleus, DMH and PVN show galanin immunocytochemical staining [85]. When injected icv, or directly into the PVN, VMH and lateral hypothalamus, galanin is mildly orexigenic, perhaps by facilitating NPY's actions [85,86]. Galanin blocks arcuate neuronal firing in those neurones expressing *gal-r1 *receptor mRNA, perhaps via direct contact with arcuate nucleus POMC neurones [87].

## POMC neurones and the brainstem

POMC neurones in the NTS, expressing GFP, are activated both by cholecystokinin (CCK) and by satiety induced by feeding, as shown by immunocytochemical measurement of Fos protein [88]. The MC3R/MC4R antagonist SHU9119 administered icv prevents CCK-induced suppression of feeding, whether administered into the third or fourth ventricle [88]. This effect of CCK activating POMC "GFP" NTS neurones is blocked by a CCK antagonist and also antagonised by endogenous opioids [89]. The dorsal motor nucleus of the vagus nerve (DMX), lying next to the NTS, has the highest density of MC4R in the brain [90] and may mediate the anorexia due to activation of the NTS POMC neurones by CCK [88]. The DMX also receives fibres from the arcuate nucleus POMC neurones, as shown by immunocytochemistry (Table 1; [91]). Vagotomy results in a slight reduction in food intake, but no change in body weight. This is associated with activation on neurones in the NTS, as well as increased AgRP and decreased POMC mRNA expression in the hypothalamus [92]. Using immunocytochemistry to demonstrate both POMC and AgRP fibres and in situ hybridisation to show their MC4R and MC3R targets (Table 3), much of the neuroanatomy of the melanocortin system has been mapped [93]. Interestingly, there are no AgRP neurones projecting to the POMC neurones in the brainstem [93]. The role of POMC neurones in the NTS and vagal afferents to both sets of POMC cell bodies requires further study.

**Table 3:** MC4R mRNA and protein expression and MC3R mRNA expression in CNS regions involved in appetite regulation. Potential targets for POMC and AgRP neurones (references indicated numerically).

**MCR locations:**	**MC4R mRNA**	**MC4R GFP expression**	**MC3R mRNA**
**Arcuate nucleus**	90	105	93, 114, 118
**Brainstem**	90	105	-
**PVN**	90, 93	105	118
**Lateral hypothalamus**	90	105	118
**DMH**	90	105	118
**SON**	90	-	-
**VMH**	90	105	118
**Periventricular nucleus**	90	105	118
**Nucleus accumbens**	90	105	-
**Amygdala**	90	105	118

### Leptin, POMC and the brainstem

LEPRB is found in several brainstem nuclei involved in the control of food intake, such as the DMX, area postrema, NTS, parabrachial, hypoglossal, trigeminal, lateral reticular and cochlear nuclei, locus coeruleus and inferior olive [94]. Leptin injected into either the fourth or lateral ventricles, or into the dorsal vagal complex (DMX, AP and NTS), suppresses feeding [94]. This suggests that the brainstem neurones are just as effective at mediating anorexia as the hypothalamic neurones and, like POMC, are direct targets for the action of leptin in the control of energy homeostasis [94]. However, part of this effect may be being mediated by hypothalamic arcuate nucleus POMC cells, as retrograde tracing experiments showed that a small percentage of these hypothalamic neurones project to the dorsal vagal complex [91].

Also, when leptin is administered centrally it activates the arcuate POMC neurones, but not the NTS neurones [95]. Recently, leptin has been shown to modulate taste sensation directly, via the taste receptors, as well as via the brainstem [96]. Leptin could have a refining influence on feeding, via its actions on taste receptors and brainstem nuclei, but this is unlikely to involve brainstem POMC neurones in the process.

## MC4R and MC3R; feeding and obesity

### MC4R, feeding and obesity

In contrast to the restricted distribution of the POMC neurones, MC4R and MC3R are widely present throughout the brain, although with different patterns of distribution (Table 3). The evidence, either genetic or neuropharmacological, for the key role of MC4R in feeding and the pathogenesis of obesity is as follows.

The most common monogenic forms of human obesity are *MC4R *mutations [7]. Murine and human *MC4R *homozygous mutants are obese and hyperphagic [97-100]. Murine and human *MC4R *heterozygous mutants are obese to a lesser extent. This shows sensitivity to quantitative variation in *MC4R *expression [97,100], with mechanisms such as poor cell-surface expression or intracellular retention of the mutant receptors [101,102]. It is also possible that genetic defects in the intracellular trafficking mechanisms, required to present MC4R on the cell surface, could also lead to human obesity [103]. Interestingly, human *MC4R *gene variants have also been associated with a lack of physical activity [104].

*mc4r *mRNA and MC4R protein is concentrated in the feeding areas of the PVN, DMH and lateral hypothalamus [90,105]. The obesity of the lethal yellow and viable yellow strains of mice is due to over-expressed agouti (with AgRP-like actions) in the hypothalamus, having an antagonistic effect at the MC3R and MC4R receptor [106]. MC4R agonists reduce feeding in rodents, with antagonists having the opposite effect [107,108]. Finally, MC4R agonists administered intranasally decrease bodyweight in humans [109]. Also simple behavioural change, such as scheduling meal times, can significantly reduce the anorexic effect of MC3R/MC4R agonists in rats [110]. Perhaps the MC4R receptor does integrate the anorexigenic signal from leptin via the POMC neurones of the arcuate nucleus and brainstem. However, it is unlikely to be the only pathway. Double mutant *ob/ob mc4r/mc4r *ko mice show partial leptin resistance when compared with *ob/ob *strains, suggesting an alternate central anorexic signalling system for leptin exists [111].

POMC neurones in the arcuate nucleus, acting on other feeding areas such as the PVN via MC4R activation, may be the principal CNS conduit for leptin's peripheral satiety signal.

### MC3R, intra-arcuate connections and obesity

Similarly one can review the evidence, either genetic or neuropharmacological, for the function of MC3R in the pathogenesis of obesity.

*mc3r *ko mice are obese with increased fat mass and decreased lean body mass, but without hyperphagia, in contrast to *mc4r *ko mice. However, mice lacking both *mc3r *and *mc4r *are more obese than *mc4r *ko mice alone [112,113]. Also, the obesity of *mc3r *ko mice is more dependent on fat intake than that of the *mc4r *ko mice [114]. Diet induced obesity in these two ko strains affects insulin-sensitivity more adversely in the *mc4r *ko mice [114]. *mc4r *ko mice do not respond to the anorectic action of MTII [73]. *MC3R *gene variants are common in humans, but they are not associated with obesity [115]. However, MC3R may mediate different responses to leptin than MC4R. While leptin administration reduces food intake in *mc4r *ko mice, *mc3r *ko mice do not show an anorexic response to leptin. This suggests that the ability of leptin to reduce food consumption depends more upon MC3R, rather than MC4R [116].

The MC3R is particularly expressed on the arcuate nucleus, including on the POMC/CART neurones [117,118] and the AgRP/NPY neurones [61] and is also more generally expressed in the CNS than the MC4R [90,118]. α-MSH, β-MSH and γ_2_-MSH all activate different neuronal targets within this nucleus [42]. However, it should be noted that MC4R is found on the arcuate nucleus too [90,105]. There are intra-arcuate POMC connections, suggesting that MC3R may mediate an autofeedback mechanism in the arcuate nucleus (Tables 1 and 3; [61,118,119]). Administration of a specific MC3R agonist reduces the frequency of action potentials in POMC-containing neurones in the arcuate nucleus, which supports this hypothesis [62]. It is not yet known if any of these observations are important with regard to feeding behaviour, but they may be important with regard to overall control of POMC neural projections. Overall, the role of MC3R in feeding behaviour and obesity is less clear than for MC4R.

## Proopiomelanocortin products and feeding

It is clear that one or more of the POMC products is involved in the anorectic response, as mice lacking either the whole coding region of POMC or the whole POMC gene are obese [8,9]. While POMC is the precursor for at least three melanocortin peptides, α-, β- and γ_2_-MSH (Figure 1), it has been widely assumed that α-MSH is the predominant ligand involved in appetite regulation in mammals, apart from humans [41,120]. β-MSH is not produced by rodents [7,120]. One impediment to comparative studies of melanocortin function is that it is very difficult to distinguish between the MSHs by immunocytochemistry, because of their similarity in core structure. Also, POMC neurones may release more than one peptide at any given synapse [42]. Finally, unprocessed POMC is found in human cerebrospinal fluid (CSF), at a concentration 100 fold higher than that of ACTH and may have a signalling role in the brain as well [121]. Obese leptin resistant *fa/fa *rats and fasted (low leptin) wild type rats have lower CSF levels of POMC than fed rodents [122]. It should be noted that as leptin regulates POMC synthesis and release, it is difficult to dissect the role of POMC turnover in the CSF [122].

### α-MSH and desacetyl-α-MSH

α-MSH suppresses feeding in free-feeding or fasted rodents, when administered centrally, as do its synthetic analogues containing the core MCR binding sequence [42,106,123,124]. Desacetyl-α-MSH, in which the N-terminal serine remains unacetylated, is a major precursor of α-MSH and is found widely in the brain [125]. Whilst not being shown to activate hypothalamic neurones in vivo [42], it binds to and activates both MC3R and MC4R in vitro and has an anorectic effect at high doses in vivo [123]. Leptin facilitates the acetylation of desacetyl-α-MSH to the more active melanocortin, α-MSH [126]. Perhaps a failure to acetylate desacetyl-α-MSH in mammals could lead to obesity? This has not yet, however, been demonstrated.

### β-MSH and γ-MSH

In radioligand binding studies, β-MSH has a higher affinity for the MC4R than α-MSH, with γ-MSH having the lowest affinity. In contrast, γ-MSH binds to the MC3R with higher affinity than either α-MSH or β-MSH [127]. It should be noted that binding of a ligand to a receptor does not necessarily correlate with biological activation of the cell expressing the receptor. Two independent studies have shown that β-MSH can have a suppressive effect on feeding in rats free-feeding or fasted for 24 hours [123,124], but this has not been found in one study looking at rats fasted for 48 hours [42]. Perhaps this is because β-MSH is not produced physiologically in rodents [7,120] and so any observed effects could be through pharmacological activation of the MC4R? [123,124,127] In a recent study in ko mice lacking *pomc*, central administration of α-MSH, β-MSH and γ-MSH can all reduce food intake, but only α-MSH actually reduces weight gain and significantly reverses the obese phenotype of the ko mouse [120]. A selective β-MSH-derived peptide agonist has been shown to decrease food intake and weight gain in diet-induced obese rats, but not MC4R-deficient mice [128]. This suggests that the anorectic pharmacological action of this β-MSH agonist in vivo is via the MC4R [128]. Recently, several missense mutations have been identified in human *β-MSH*, associated with early-onset human obesity [41,129], as well as a cleavage site mutation preventing processing of POMC to β-MSH, which is also associated with human obesity [40]. γ_2_-MSH has a delayed anorectic effect in fasted rats [42]. These data are consistent with MC4R being the principal anorectic receptor in mammals, with either α-MSH or β-MSH being the principal ligands depending upon species [7,41,120]. Perhaps the delayed feeding effect of γ_2_-MSH reflects autostimulation of the POMC neurones projecting to the PVN, where POMC products bind to MC4R? Alternatively, it could reflect an inhibitory action via MC3R on NPY neurones in the arcuate nucleus [61]. It is possible that individual arcuate POMC projections in mammals release different combinations of peptides (MSHs or β-endorphin) at the different hypothalamic and extra-hypothalamic sites with which they synapse, as part of the complex integration of feeding behaviour in the CNS [42].

### Melanocortin-opioid interactions

β-endorphin is another post-translational product of POMC and, together with the opioid peptides, the enkephalins and dynorphin, acts at μ- and κ-receptors to stimulate feeding [130-133]. Opioid antagonists block NPY induced feeding [134]. *β-endorphin *ko mice gain 10–15% more body weight than wild type after puberty [135]. Thus, there may be a more complementary interaction between the various POMC peptides in the regulation of feeding [136]. For example, it is possible that secretory vesicles could contain both MSHs and β-endorphin, which would have antagonistic effects on feeding. Immunocytochemistry has been used to show that there are synaptic connections between POMC and enkephalin neurones in the arcuate nucleus [137]. Using techniques already described in this article, the nucleus accumbens has been shown to have a POMC projection from the arcuate nucleus (Table 1; [93]). It also contains endogenous opioids which mediate the positive emotional response to palatable foods such as sugar and fat. This may be their main role in appetite control, as opioid-evoked feeding is generally short lived [138].

## POMC and the paraventricular nucleus of the hypothalamus

The PVN has both neuroendocrine and feeding roles. It has two major subdivisions, magnocellular and parvocellular, that fan either side of the roof of the 3rd ventricle. There is immunocytochemical evidence that many of the POMC neurones in the arcuate nucleus project to the PVN (Table 1; [93]) where the *mc4r *mRNA is present in abundance (Table 3; [90]). Surgical disruption of this pathway leads to obesity [58]. Activation of MC4Rs decreases body fat stores by reducing food intake and increasing energy expenditure. To identify which sites of *mc4r *expression are most relevant for mediating these effects, mice were generated with a loxP-modified, null *mc4r *allele (loxTB *mc4r*) that can be reactivated by Cre-recombinase [139]. Mice homozygous for the loxTB *mc4r *allele do not express MC4Rs and are very obese [139]. Restoration of normal *mc4r *expression in the PVN and a subpopulation of amygdala neurones, using s*im1*-Cre transgenic mice, prevented 60% of the obesity [139]. Notably, increased food intake was completely rescued while reduced energy expenditure was unaffected. These experiments suggest that MC4Rs in the PVN and/or the amygdala control food intake, but that MC4Rs elsewhere control energy expenditure [139]. All three MSHs, plus MTII, induce IEG expression in this area, although it is not known to what extent this reflects direct actions of the melanocortins (e.g., α-MSH and β-MSH) within the PVN or indirect activation (e.g., γ_2_-MSH) via the MC3R on the arcuate nucleus POMC neurones projecting to the PVN [42,140]. The PVN expresses LEPRB and is thus responsive to leptin [18,19]. Rats overfed postnatally, who are hyperleptinaemic, acquire altered electrophysiological responses in the PVN to α-MSH, AgRP, MTII, CART, melanin-concentrating hormone (MCH) and NPY respectively [141]. Injection of α-MSH, but not β-endorphin, into the PVN reduces POMC gene expression in the arcuate nucleus, implying a negative feedback system involving melanocortins only [142].

### Nesfatin-1, α-MSH and the PVN

Nesfatin-1 is a novel satiety molecule that is processed from the nucleobindin2/nesfatin precursor molecule [143,144] and its mRNA is distributed in hypothalamic nuclei including the arcuate nucleus and PVN [145]. Nesfatin-1 concentrations are reduced in the PVN with fasting. Central administration of leptin does not alter nucleobindin2/nesfatin mRNA expression. Also, the satiety effect of nesfatin-1 is not altered in Zucker rats with an *leprb *mutation and prior administration of an anti-nesfatin-1 antibody does not block leptin-induced anorexia. This implies that leptin signalling is independent of nesfatin-1 signalling. In contrast, central administration of α-MSH markedly stimulates nucleobindin2/nesfatin gene expression in the PVN [145]. Prior administration of the MC3R/MC4R antagonist SHU9119 abolishes nesfatin-1-induced feeding suppression, but nesfatin-1 does not show any direct agonistic action on MC3R or MC4R. These observations suggest that nesfatin-1 signalling is involved in a leptin-independent melanocortin signalling pathway in the hypothalamus.

It is clear that the arcuate nucleus to PVN circuit is fundamental in the transmission of the satiety message in the hypothalamus.

### Neuroendocrine targets for POMC products in the PVN

Significant proportions of neurones activated by α-MSH, β-MSH and γ_2_-MSH project outside the BBB and are thus presumed to be neuroendocrine in origin [42], such as CRF and TRH. Fasting suppresses CRF release, which is blocked by α-MSH [146] and melanocortins can induce CRF gene transcription in some PVN CRF nerves expressing MC4R [147]. This suggests that CRF acts downstream of the melanocortin system. TRH release is elicited by α-MSH, whereas γ-MSH and AgRP inhibit TRH release. MC4R and MC3R agonists mimic the effect of α-MSH and γ-MSH respectively [148]. This implies that hypothalamic TRH release is controlled by POMC and AgRP neurones. In the POMC ko mouse, there is reduced pituitary TSH and hypothalamic TRH production, but the thyroid gland compensates, with elevated plasma T_3 _and T_4 _[149]. These central effects of the melanocortins on the stress and thyroid axes suggest that POMC neurones may influence peripheral metabolism, which will in turn indirectly affect feeding behaviour.

## POMC and the lateral hypothalamus

Damage to the lateral hypothalamus inhibits feeding, reducing body weight [150]. The lateral hypothalamus expresses *leprb *mRNA and is regulated by leptin throughout development [151]. Consistent with the anorexigenic role of hypothalamic POMC pathways, α-MSH, β-MSH and γ_2_-MSH activate neurones in the more central hypothalamic structures that are associated with satiety, rather than in the lateral hypothalamus, which has an established role in feeding [42,152]. However, both *mc3r *and *mc4r *mRNA are located in the lateral hypothalamus (Table 3; [90,118]) and there is immunoreactive evidence of both POMC and AGRP fibres projecting from the arcuate nucleus to the lateral hypothalamus (Table 1; [93]), so the lack of response to the MSHs administered icv is perhaps surprising.

### MCH and POMC

MCH causes hyperphagia, whether injected icv or directly into the arcuate nucleus, PVN and DMH. This is achieved in part by altering the balance of neuronal activity, favouring NPY/AgRP release and reducing α-MSH/CART release from arcuate neurones [152-154]. For example, in hypothalamic explant experiments injection of MCH into the hypothalamus increased the production of NPY and AgRP and decreased the production of MSH and CART, as measured by radioimmunoassay [153].

### Orexins and POMC

Orexin expressing cells are located in the lateral, dorsal and perifornical nuclei. They innervate the arcuate nucleus, preoptic area, paraventricular nucleus of the thalamus, septal nuclei, locus coeruleus and DMX in the brainstem, as measured immunocytochemically. There is also immunocytochemical evidence that orexin neurones synapse with NPY/AgRP and POMC/CART neurones in the arcuate nucleus. Microinjection of orexins into the arcuate nucleus, PVN and lateral hypothalamus stimulates feeding [155-157]. It is possible to measure the action potential of individual living arcuate POMC neurones in mouse brain slices, identified by GFP transgenic tagging. Using whole cell patch clamp recordings, orexin suppresses the spontaneous firing in these neurones, suggesting that its appetite enhancing effects include an effect in suppressing hypothalamic POMC neuronal activity [158].

## POMC and the dorsomedial nucleus of the hypothalamus

The DMH is an important leptin target, as it expresses both *leprb *mRNA and protein [18,19]. Lesioning experiments suggest that it has extensive projections to the PVN, particularly portions involved in autonomic control [159]. These experiments also show that lesions of this nucleus produce hypophagia and reduce linear growth [159]. Using techniques already described in this article, there is evidence of POMC projections from the arcuate nucleus to the DMH (Table 1; [93]) and both *mc3r *and *mc4r *mRNA are found in this nucleus (Table 3; [90,118]). Using a retrograde-labelled approach to measure axonal transport, there is evidence that some of these projections from the DMH to the PVN may be under the control of POMC neurones originating in the arcuate nucleus [160].

Despite the presence of the MC3R, only α-MSH and β-MSH induce IEG expression in the DMH when administered icv. Very few of these neurones, which demonstrate increased IEG activity, project outside the BBB [42]. In *mc4r *ko mice, or lethal yellow mice overexpressing agouti, NPY expression (but not galanin or POMC) is significantly elevated in the DMH. This suggests that POMC may normally inhibit NPY expression tonically in the DMH and that AgRP may be a determinant of NPY release physiologically [161]. The DMH has direct brainstem projections to the DMX, which has the highest density of MC4R in the brain [90,162].

## POMC and the supraoptic nucleus of the hypothalamus

The SON lies laterally in a bilateral position at the base of the hypothalamus. The SON and the mPVN contain oxytocin and vasopressin cells, which project to the posterior pituitary, where these hormones are released [3,163]. All three MSHs activate IEGs in the SON and coexpression with fluorogold suggests that all of these targets activated by α-MSH and γ_2_-MSH, as well as the vast majority of those activated by β-MSH, project outside the blood-brain barrier in the rat [42]. β-MSH is not a physiological ligand of the MC4R in rodents which may explain this partial effect [42]. The effect of γ_2_-MSH is rather surprising, given the presence of the MC4R and the absence of the MC3R in this nucleus (Table 3; [42]). Again, this may reflect indirect activation, perhaps via an arcuate nucleus POMC neuronal projection to the SON, as evidenced by immunocytochemical detection of POMC fibres between these nuclei and in situ hybridisation for MC3R in the arcuate nucleus and MC4R in the SON (Table 1; [90,93,118]). CCK co-expression in oxytocin neurones in the SON occurs in rats on a normal diet only, rather than a high fat diet [164]. MC4R alone is expressed in the SON and MC4R ko mice are particularly prone to gaining weight on a high fat diet, even when compared with *ob/ob *mice [165]. Perhaps POMC products acting on the MC4R, under dynamic antagonism from AgRP [93], form part of the CCK pathway in the SON involved with fat intake?

## POMC and the ventromedial nucleus of the hypothalamus

The VMH lies close to the DMH, arcuate nucleus and third ventricle. The VMH has no known neuroendocrine projections [42], but lesioning experiments show that it is involved in feeding behaviour in the rat [166]. The projection from the arcuate nucleus to the VMH is very sparse and very few of these fibres are POMC neurones on immunocytochemistry (Table 1; [93]). Despite this, *mc3r *and *mc4r *mRNA are abundant (Table 3; [90,118]). MTII increases neuronal firing in the VMH in vitro and this effect is lessened by prior food deprivation [167]. Perhaps free POMC in the CNS has a true neuroendocrine role and is post-translationally processed locally by the VMH? [121] This could explain the extensive expression of *mc3r *and *mc4r*, despite the very limited POMC axonal presence in the VMH.

### Brain-derived neurotrophic factor, TrkB and POMC neurones

Brain-derived neurotrophic factor (BDNF) and its receptor, TrkB, control neurodevelopment and synaptic plasticity. *TrkB*-deficient humans show learning difficulties and severe obesity [168]. *bdnf*-deficient heterozygous mice are obese, with high leptin and insulin levels which correct with diet alone [169]. *bdnf *mRNA or protein is expressed in the VMH, DMH and lateral hypothalamus, but not the arcuate nucleus [169,170]. Its expression is reduced in the VMH of lethal yellow mice, whereas administration of MTII increases BDNF expression in the VMH of wild type mice. BDNF suppresses feeding and weight gain in MC4R ko mice, so its anorexic actions lie downstream of POMC. It is unlikely that BDNF modulates either CART/POMC or NPY/AgRP neurones in the arcuate nucleus as there is no *trkb *receptor mRNA expressed in this nucleus [170].

## POMC and the periventricular nucleus of the hypothalamus

Cafeteria diet-induced obese rats have been shown to have increased somatostatin protein content and gene expression in the periventricular nucleus [171]. Using a double-labelled immunocytochemical approach, arcuate nucleus POMC neurones have been shown to project to the majority of somatostatin perikarya in the periventricular nucleus [172]. This implies that the effects of somatostatin on growth and body composition are under the influence of the melanocortins [172]. Similarly, somatostatin receptors are located on POMC arcuate nucleus neurones, suggesting bidirectional communication [172]. These observations link the hypothalamic functions of appetite and growth regulation.

## POMC, the nucleus accumbens and the amygdala

There is immunocytochemical evidence that the nucleus accumbens of the forebrain receives both POMC and AgRP projections from the arcuate nucleus (Table 1; [93]), as well as a dopamine projection which mediates the reinforcing effects of stimulants, like cocaine, amphetamine, nicotine and caffeine [173-175]. Microinjection of muscimol, a GABA_A _receptor agonist, into the nucleus accumbens, increases feeding. This is associated with activation of orexin neurones (but not MCH) in the lateral hypothalamus and the activation of the NPY/AgRP projection and inhibition of the POMC/CART projection in the Arc [176]. This may be a mechanism for the appetite modulating effects of cocaine, amphetamine, nicotine and caffeine.

The amygdala, located in the mid-temporal lobe, assigns emotional significance to sensory information, such as fear-related responses [177]. It receives auditory and other efferents from the thalamus, cortex and hypothalamus. In turn, it projects to brainstem and hypothalamic regions that regulate autonomic, endocrine and feeding responses [178]. For example, there is evidence from immunocytochemistry that it receives POMC/CART and AgRP/NPY projections from the arcuate nucleus (Table 1; [93]). The release of CRF and bombesin-like peptides in the amygdala is markedly increased by both stress and feeding [179]. These CRF and bombesin amygdala neurones could be influenced by arcuate POMC or AgRP projections to the amygdala triggered by either stress or alteration in nutritional balance [93].

## Discussion

Polygenic traits, such as obesity, result from complex combinations of multiple contributing genes [180], environmental factors [181], or gene-environment interactions [182]. In 1980, 8% of women and 6% of men in England were obese, with a body-mass index greater more than 30 kg/m^2^. By 1998, the proportion of obese subjects had increased to 21% of women and 17% of men, suggesting perhaps that environment is a key factor [183]. However, several rare monogenic human obesity syndromes have recently been described. These include deficiencies of leptin, *LEPRB*, single-minded 1 (*SIM1*), *PC1*, *POMC *and *MC4R *deficiency [184]. All of these syndromes are associated with hyperphagia. The last three of these gene defects produce altered protein products involved in POMC neuronal transmission (Figure 1). The other three are key factors interacting with the central melanocortin pathway. Leptin binding to LEPRB is the main peripheral hormonal stimulus to arcuate POMC neurones [11] and SIM1 is specifically involved in the development of the PVN [185]. The PVN is the destination of the principal arcuate POMC projection involved in suppressing feeding [47,62,93]. *sim1 *heterozygous deficient mice have a similar phenotype to MC4R deficient and lethal yellow mice, with obesity resistant to the effects of melanocortins [186]. Also, when the melanocortin antagonist AgRP is mutated in either rodents or humans, it leads to thinness [53-55]. Thus, there is considerable human and murine genetic evidence for the involvement of the central melanocortin pathways in the control of appetite [7]. Could genetic variation in this system (and that of related interacting molecules) be part of the polygenic tendency to obesity in humans [7,40,41,44-46,129,180,184]?* MC4R *deficiency is the commonest of all the human single-gene defects causing obesity, responsible for 5% of all severe cases [7]. *POMC *heterozygosity has also been found to correlate with obesity in mice and humans, to a lesser extent than the homozygous state [9,187]. POMC neurones may mediate a central anorexic signal in proportion to body adipocyte reserves, possibly via the actions of β-MSH on the MC4R in humans [41,129].

## Conclusion

In summary, the likely model for the melanocortin regulation of feeding is that the AgRP signal in the arcuate nucleus fluctuates to modulate a more constant POMC signal [57,59], which is a function of the relatively steady hormonal level of leptin [49]. POMC products may be released differentially in the different hypothalamic and extra-hypothalamic sites involved with feeding in the CNS [42], which could lead to subtle variations in the anorexic signal being transmitted. This may or may not be independent of leptin signalling.

## Competing interests

The author(s) declare that they have no competing interests.

## Authors' contributions

GWMM conceived and wrote this review article. The author read and approved the final manuscript.
